# Supplementary material for: Comparative analysis of early ontogeny in Bursatella leachii and Aplysia californica
Source: PeerJ. 2014 Dec 11;2:e700. doi: 10.7717/peerj.700 (PMC4266853; doi:10.7717/peerj.700)
Supplement: Supplemental Information 1 — Veliger shell length of A. californica and B. leachii larvae grown at 25 °C and A. californica larvae grown at 22 °C and 25 °C in 2006. Shell length was measured weekly from day of hatching until 80% competency, error bars represent ±1 standard deviation. Values are the number of days post-hatching until the specified developmental stage was observed. [file peerj-02-700-s001.docx]

**Supplementary 1 – Comparison of larval and juvenile growth of *Bursatella leachii* and *Aplysia californica* in laboratory settings.**

Veliger shell length of *A. californica* and *B. leachii* larvae grown at 25˚C and *A. californica* larvae grown at 22˚C and 25˚C in 2006. Shell length was measured weekly from day of hatching until 80% competency, error bars represent ± 1 standard deviation. Values are the number of days post-hatching until the specified developmental stage was observed.

|  | Age (dph) | Stage | Mean SL (μm) | SL  Stdev (μm) |
| --- | --- | --- | --- | --- |
| *Bursatella leachii* @25˚C | 0 | 1 | 141.1 | 6.9 |
|  | 5 | 2 | 264.6 | 13.9 |
|  | 7 | 4 | 284.2 | 19.0 |
|  | 13 | 7 | 290.1 | 12.4 |
|  | 15 | 8 | 290.1 | 8.3 |
| *Aplysia californica* @25˚C | 0 | 1 | 134.6 | 3.7 |
|  | 7 | 2 | 236.1 | 18.6 |
|  | 14 | 3 | 360.6 | 36.9 |
|  | 21 | 5 | 392.3 | 18.9 |
| *Aplysia californica* @22˚C | 0 | 1 | 134.6 | 3.7 |
|  | 7 | 2 | 227.6 | 15.0 |
|  | 14 | 3 | 337.7 | 20.8 |
|  | 21 | 5 | 392.8 | 10.0 |
