# Supplementary material for: Comparative analysis of early ontogeny in Bursatella leachii and Aplysia californica
Source: PeerJ. 2014 Dec 11;2:e700. doi: 10.7717/peerj.700 (PMC4266853; doi:10.7717/peerj.700)
Supplement: Supplemental Information 2 — Mean shell length (mean ± standard deviation (Stdev)) of Aplysia californica larvae grown at 22 °C and 25 °C for stage 1, 2, 3 and 5 in 2006. Mean shell length at 22 °C for each stage n = 25 shells; Mean shell length at 25 °C for each stage n = 25. Two-way Repeated Measures ANOVA, 18 df, p < 0.0001. [file peerj-02-700-s002.docx]

**Supplementary 2 – Comparison of larval and juvenile growth of *Aplysia californica* in laboratory settings at** **22**˚**C and 25** ˚**C.**

Mean shell length (mean ± standard deviation (Stdev)) of *Aplysia californica* larvae grown at 22˚C and 25˚C for stage 1, 2, 3 and 5 in 2006. Mean shell length at 22˚C for each stage n=25 shells; Mean shell length at 25˚C for each stage n=25. Two-way Repeated Measures ANOVA, 18 df, p<0.0001.

**
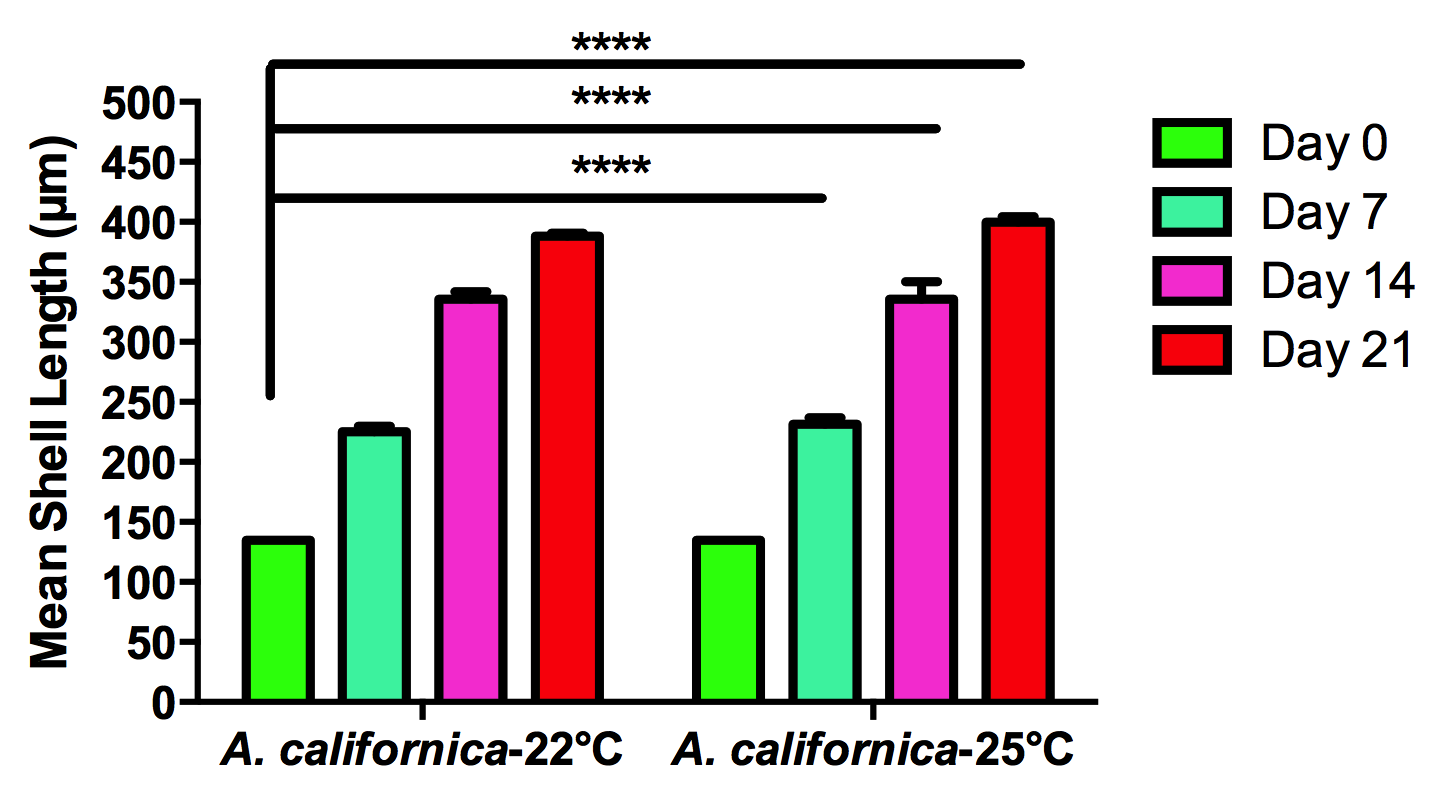
**
