# Supplementary figures and images for: Comparative analysis of early ontogeny in Bursatella leachii and Aplysia californica
Source: PeerJ. 2014 Dec 11;2:e700. doi: 10.7717/peerj.700 (PMC4266853; doi:10.7717/peerj.700)

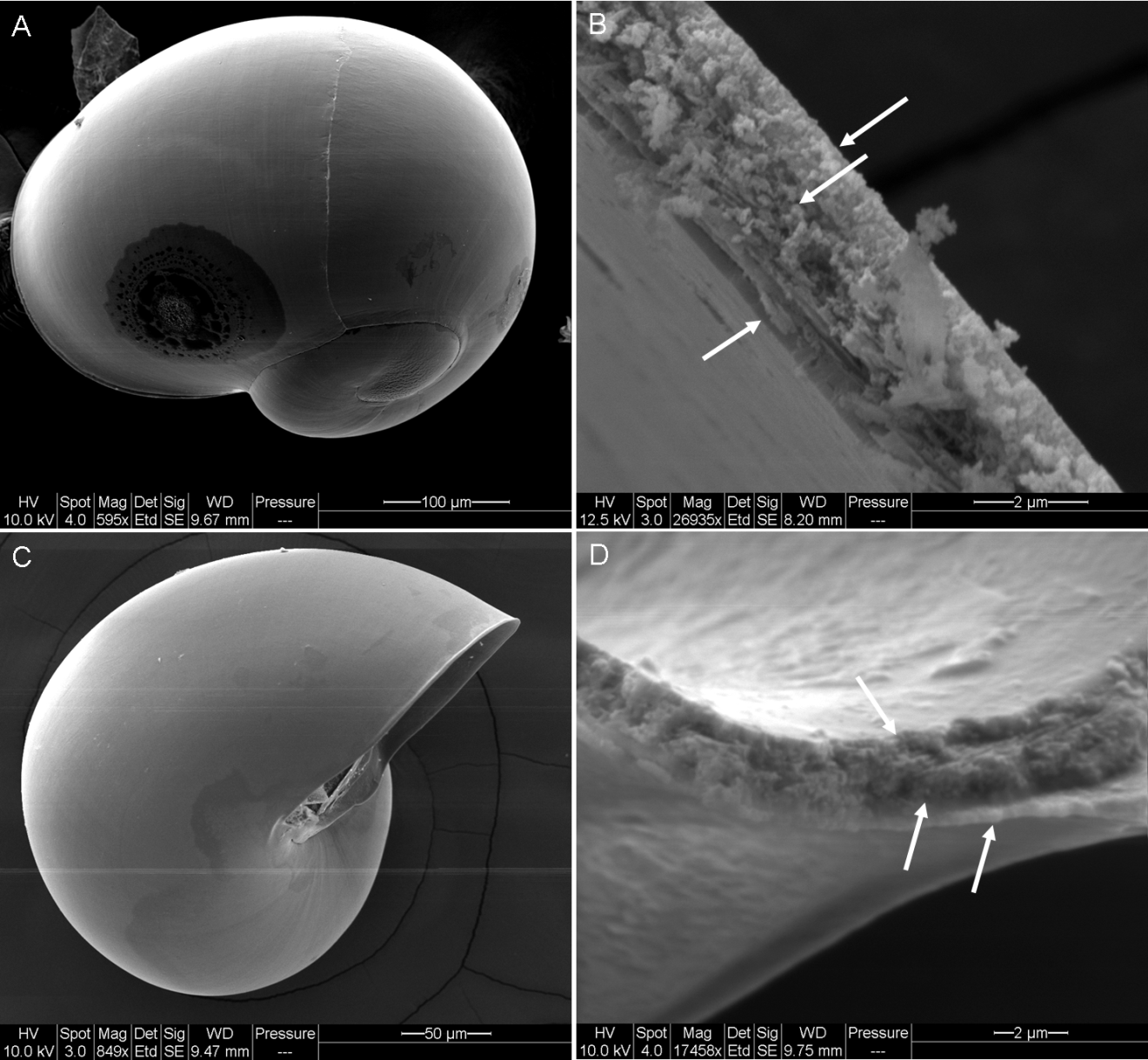

Supplement: Supplemental Information 3 — Whole shell of Stage 6 veligers of Aplysia californica (A) and Bursatella leachii (C) and cross sections of A. californica (B) and B. leachii (D) [file peerj-02-700-s003.png]
